# Geographic Spatial Distribution Patterns of *Dirofilaria immitis* and *Brugia pahangi* Infection in Community Dogs in Chiang Mai, Thailand

**DOI:** 10.3390/ani11010033

**Published:** 2020-12-26

**Authors:** Manusvee Kaikuntod, Orapun Arjkumpa, Doolyawat Kladkempetch, Shinya Fukumoto, Kriangkrai Thongkorn, Chavalit Boonyapakorn, Veerasak Punyapornwithaya, Saruda Tiwananthagorn

**Affiliations:** 1 Department of Companion Animals and Wildlife Clinic, Faculty of Veterinary Medicine, Chiang Mai University, Chiang Mai 50100, Thailand; manusvee.k@gmail.com (M.K.); kktk23@hotmail.com (K.T.); chavalitboonyapakorn@gmail.com (C.B.); 2 Phra Nakhon Si Ayutthaya Provincial Livestock Office, Thanu Subdistrict, Uthai District, Phra Nakhon Si Ayutthaya 13000, Thailand; arjkumpa@hotmail.com; 3 Department of Veterinary Biosciences and Veterinary Public Health, Faculty of Veterinary Medicine, Chiang Mai University, Chiang Mai 50100, Thailand; doolyawat_kl@cmu.ac.th; 4 National Research Center for Protozoan Diseases, Obihiro University of Agriculture and Veterinary Medicine, Obihiro 080-8555, Hokkaido, Japan; fukumoto@obihiro.ac.jp; 5 Department of Food Animal Clinic, Faculty of Veterinary Medicine, Chiang Mai University, Chiang Mai 50100, Thailand; 6 Research Group for Veterinary Public Health, Faculty of Veterinary Medicine, Chiang Mai University, Chiang Mai 50100, Thailand; 7 Research Center of Producing and Development of Products and Innovations for Animal Health and Production, Faculty of Veterinary Medicine, Chiang Mai University, Chiang Mai 50100, Thailand

**Keywords:** *Brugia pahangi*, *Dirofilaria immitis*, PCR-RFLP, community dogs, spatial distribution, altitude

## Abstract

**Simple Summary:**

Filariasis is emerging as a public health concern for humans, dogs, cats, and other wildlife species, and is frequently found in southeast Asian countries. The present study confirmed the species of filarial nematodes in free-roaming dogs from temple communities. Two species were found: *Dirofilaria immitis* infection and, for the first time, *Brugia pahangi*. The occurrence of the two species was comparable. Geographic spatial distribution revealed the abundance of *D. immitis* and *B. pahangi* in the central areas at altitudes less than 400 m. However, at higher altitudes between 400 and 800 m, we found a significantly higher number of *B. pahangi* infections than *D. immitis* infections. In conclusion, *D. immitis* and *B. pahangi* were the most common filarial infections found in community dogs in Northern Thailand. Dogs might be an important reservoir for *B. pahangi* in that region. The population dynamics of the mosquito vector of *B. pahangi* across altitudinal gradients merits further study.

**Abstract:**

Filariasis is emerging as a public health concern in tropical and subtropical areas. Filariasis is an endemic problem commonly found in southeast Asian countries. Using the PCR-restriction fragment length polymorphism (PCR-RFLP) of the ITS1 region with *Vsp* I, the overall prevalence rates of *Dirofilaria immitis* (12.2% (41/337); 95% confidence interval: 9.1–16.1%) and *Brugia pahangi* (8.3% (28/337); 95% confidence interval: 5.8–11.8%) were determined based on 337 free-roaming community dogs from 20 districts in Northern Thailand. Microfilaremia was found in only 6.2% of dogs (21/337). Co-infection with *D. immitis* and *B. pahangi* was observed in two dogs. Of the 215 blood samples examined using a Canine Heartworm Ag Kit, only 3.72% (eight dogs) were *D. immitis* antigen positive. Among these eight, six dogs had occult *D. immitis* infections. In terms of geographic distribution, we found the abundance of *D. immitis* and *B. pahangi* in the central areas at altitudes less than 400 m to be 12.1% and 10.3%, respectively. In contrast, at higher altitudes between 400 and 800 m, a significantly higher number of *B. pahangi* compared with *D. immitis* infected individuals were observed at 14.29% and 4.1%, respectively. In conclusion, *D. immitis* and *B. pahangi* were the most common filarial infections found in community dogs in Northern Thailand. Dogs might be an important reservoir of *B. pahangi* in that region. Increasing awareness and concern and including proper deworming programs for community dogs should be endorsed to reduce the transmission risk. Additionally, the population dynamics of the mosquito vector of *B. pahangi* across altitudinal gradients deserved further investigation.

## 1. Introduction

Filarial nematode infection is an important vector-borne disease in tropical countries. According to the World Health Organization (WHO) roadmap (WHO 2020), the goal of eliminating filariasis is expected to be achieved by 2030. Filarial infections are currently common in companion animals worldwide [1]. Specifically, in Southeast Asia, lymphatic filariasis caused by *Brugia malayi* (Brugian or Malayan filariasis), *Wuchereria bancrofti* (bancroftian filariasis) and *Brugia timori* is considered a significant human health problem. Filariasis in dogs is caused by various species, e.g., *Dirofilaria* spp., *Acanthocheilonema* spp., and *Brugia* spp. [2,3,4]. Typically, the life cycle of the filarial worm requires a bloodsucking insect such as mosquitoes as a transmission vector, known as an intermediate host. The ingested microfilariae (L1) from the dog develop into the infective stage larvae (L3) in the vector. However, the worm has a species–specific target organ within the final host [5].

Canine heartworm disease, *D. immitis* infection, is one of the serious types of filariasis observed in veterinary practice. Adult worms commonly live in pulmonary arteries and congestive heart failure can occur in severe cases. *Dirofilaria repens* and *Acanthocheilonema reconditum* are other [6] filarial worms usually located in subcutaneous tissues or subconjunctival areas. *Brugia* spp., such as *Brugia pahangi* and *Brugia malayi*, are known to cause lymphatic filariasis (LF), whereas *B. malayi* causes a serious lymphatic obstruction in humans. Whether *B. pahangi* is a causative agent of human disease in the natural environment is not yet known [7]. However, microfilaria in the blood, as well as signs and symptoms of LF, were detected in experimentally infected human volunteers [8]. A clinical description of lymphatic filariasis caused by natural infection with *B. pahangi* in Malaysia was recently published [9]. Dogs and cats are reservoir hosts of *B. malayi*, but they are the essential hosts of *B. pahangi* [10].

Currently, several methods are employed to diagnose filarial infections. The basic conventional technique generally uses microscopic examination to detect microfilariae. The microhematocrit centrifugation technique, or Woo’s test, is easy to perform, quick, and inexpensive [11,12], but it cannot identify the species of the parasites. An alternative approach for the identification of filarial worms is DNA technology. Polymerase chain reaction-restriction fragment length polymorphism (PCR-RFLP) analysis is a molecular technique that involves cutting the specific target of the PCR product with a restriction endonuclease. According to Nuchprayoon et al. [13], differentiation of a wide variety of species of filarial worms was accomplished using PCR-RFLP analysis of the internal transcribed spacer 1 (ITS1). Rishniw et al. [3] reported that using the pan-filarial primers DIDR-F1 and DIDR-R1 in a single PCR test could discriminate among six discordant microfilaria species, including *D. immitis, D. repens, A. reconditum, B. malayi,* and *B. pahangi.* The species–specific (for *D. immitis*) PCR-targeting cytochrome *c* oxidase subunit 1 (*cox1*) gene has been used to confirm *D. immitis* infection.

In tropical countries, including Thailand, the endemic areas for *D. immitis, B. malayi*, and *B. pahangi* have been reported, including different species of filarial infection [14,15]*. D. immitis* infection was found throughout Thailand and has a higher prevalence in stray dogs than in pet dogs [16]. *Brugia* spp. infection was reported to be found mostly in the southern part of Thailand [17,18]. In the Chiang Mai province in Northern Thailand, canine filariasis showed a prevalence of 18.2% in 2008; however, this study lacked reliable filarial species identification [19]. In addition, *Brugia* infection in dogs has never been confirmed in Northern Thailand. The WHO recommended using a mapping method for delimiting areas requiring mass drug treatment to save resources [20]; however, to date, no spatial clustering of vector-borne diseases of dogs has been detected in this region. The present study was aimed at updating the occurrence of filarial infection in free-roaming community dogs in the province of Chiang Mai, confirming filarial species using molecular techniques and documenting the geographic distribution of *D. immitis* and *B. pahangi* using spatial mapping in an effort to augment disease control efforts in animals and humans, thus reducing public health concerns.

## 2. Materials and Methods

### 2.1. Study Area, Sample Collection, and Ethical Concerns

**Figure 1:**
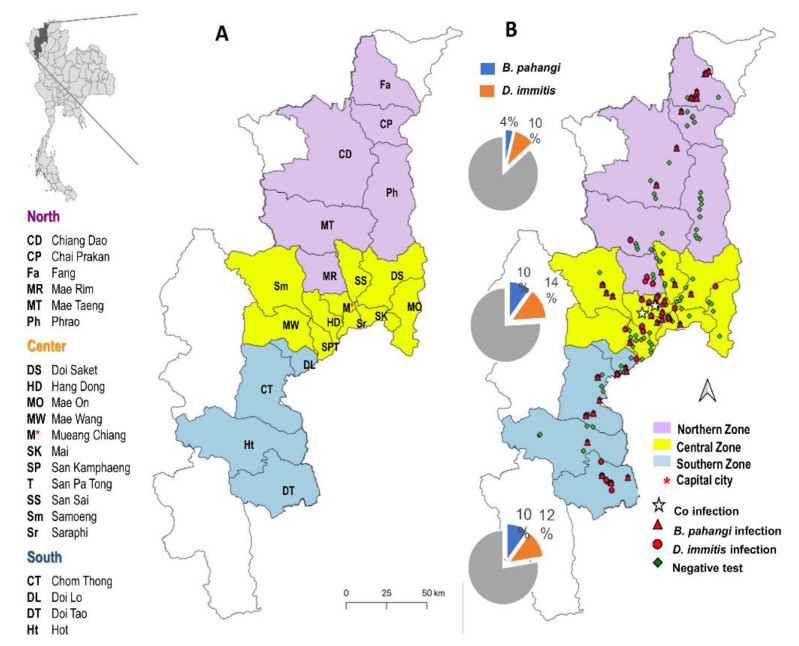
Study sites and geographic distribution of canine filariasis in three zones of Chiang Mai, Thailand. (**A**). The study districts; (**B**). Distribution of *Brugia pahangi* and *Dirofilaria immitis* positives and the proportion of prevalence of filarial nematode species in each zone.

The study area was in the province of Chiang Mai in the upper northern region of Thailand, which is considered the province with the highest dog population density in the nation (278,943 dogs in Chiang Mai of 645,368 total dogs in the region, Bureau of Disease Control and Veterinary Services 2016). The study area was divided into three zones (northern, central, and southern; Figure 1A), encompassing 146 community temples as well as households distributed over 20 of 25 districts and 72 subdistricts to include a wide range of geographical features from north to south of Chiang Mai. The 20 districts included six districts in the northern part of the province (Chiang Dao, Chai Prakan, Fang, Mae Rim, Mae Taeng, and Phrao), 10 districts in the central part (Doi Saket, Hang Dong, Mae On, Mae Wang, Mueang Chiang Mai, Samoeng, San Kamphaeng, San Pa Tong, San Sai, and Saraphi), and four districts in the southern part (Chom Thong, Doi Lo, Doi Tao, and Hot). GPS location was determined (Map Plus TM version 2.4, mobile application) for each sampling site, including coordinates (latitude and longitude) and altitude (meters). Samples were collected from June to December 2019. According to a government announcement (Thai Meteorological Department 2018), this sampling period could be defined as spanning the rainy (June to October) and cold seasons (November to February).

A total of 337 blood samples were collected from the community dogs (the dogs owned by specific owners, able to be free ranging in the community, and taken care of by people in the communities), including 168 males and 169 females. Inclusion criteria included age >7 months and having not received heartworm prevention medication. From each dog, 2 mL of blood was collected from either the cephalic or saphenous vein which was kept in EDTA tubes. The first 0.5 mL of blood was used for Woo’s examination for the presence of microfilaria. The remaining 1.5 mL was kept in a freezer at −20 °C for further molecular analysis.

All dog owners (temple master or dog keepers) signed an informed consent form, and the treatment of the animals was approved by the Animal Care and Use Committee, Faculty of Veterinary Medicine, Chiang Mai University (R15/2562) on 12 June 2019.

### 2.2. Laboratory Examination

#### 2.2.1. Detection of Circulating Microfilaria and *D. immitis* Antigens

The whole blood samples were examined for total circulating microfilariae using the microhematocrit centrifugation technique (Woo’s technique) [11]. In addition, the packed cell volume (PCV) was also measured as a percent. Measurement of the severity of anemia in this study followed the guidelines for classification of severity of anemia from the World Small Animal Veterinary Association (WSAVA) 2005 [21]. A total of 215 whole blood samples were randomly selected for additional study to detect the adult female *D. immitis* antigen using a Thinka Canine Heartworm Ag Test kit (Thinka CHW, Arkray, Kyoto, Japan) following the manufacturer’s instructions. For quality control, the blood samples were examined within 48 h after blood collection.

#### 2.2.2. Molecular Techniques and Sequencing

Genomic DNA (gDNA) was extracted from 200 µL anticoagulated blood samples. A commercial DNA extraction kit (Nucleospin^®^ Blood, Macherey-Nagel, Duren, Germany) was used following the manufacturer’s instructions.

Various diagnostic tools have been developed for the precise diagnosis of filaria infection. PCR-RFLP—which is based on a diagnostic method previously designed by Nuchprayoon et al. [13] and targets the ITS1 region (primer: ITS1-F and ITS1-R; Table 1)—was conducted. PCR amplification reactions were performed in a 20 μL reaction volume containing 100 ng gDNA (3–5 µL), 0.2 μM of each primer (0.4 μL of 10 μM), and 10 µL of 2 × Quick Taq^®^ HS DyeMix (TOYOBO, Osaka, Japan). PCR products were analyzed on a 1.5% agarose gel electrophoresis. The positive ITS1 PCR products were then digested with five units of Vsp I (SibEnzyme, Novosibirsk, Russia) as a restriction endonuclease, according to the manufacturer’s protocol. One unit of Vsp I was used to digest 1 µg of the ITS1 PCR product for 1 h at 37 °C in a total reaction volume of 50 µL. DNA fragment analysis was conducted by 1.5% agarose gel electrophoresis, stained with RedSafe™ nucleic acid staining solution (iNtRON Biotechnology, Gyeonggi-do, Korea), and visualized using a GelMax™ Imager (Ultra-Violet Products, Cambridge, UK).

Two other PCR techniques were used to confirm the presence of filarial DNA in the suspicious samples, including PCR of pan-filarial primers (DIDR-F1 and DIDR-R1) to discriminate between *A. reconditum* and *Amphiachyris dracunculoides*, and primers specific for *D. immitis* (DI COI-F1 and DI COI-R1) to confirm *D. immitis* DNA in suspected occult infections. The PCR procedure was performed following previously published methods [3] and using the same reagent previously described for the PCR of ITS1. Distilled water (DW) as a negative control and the gDNA of *D. immitis*, *B. pahangi*, and *B. malayi* as positive controls were included in the analysis.

Species confirmation in the 10 previous *D. immitis*- and *B. pahangi*-positive samples was achieved by DNA sequencing of the 5.8s-ITS2-28S amplicons using pan-filarial primers given the clear and distinctive amplicon. The PCR product (50 μL) was purified using a NucleoSpin^®^ PCR Clean-up Kit (Macherey-Nagel GmbH, Duren, Germany) and submitted for direct fluorescent dye-terminator sequencing in the sense and antisense directions by BioBasic Inc. (The Elitist, Singapore).

### 2.3. Data and Statistical Analysis

#### 2.3.1. Determination of the Presence and Prevalence of Filariasis

**Figure 2:**
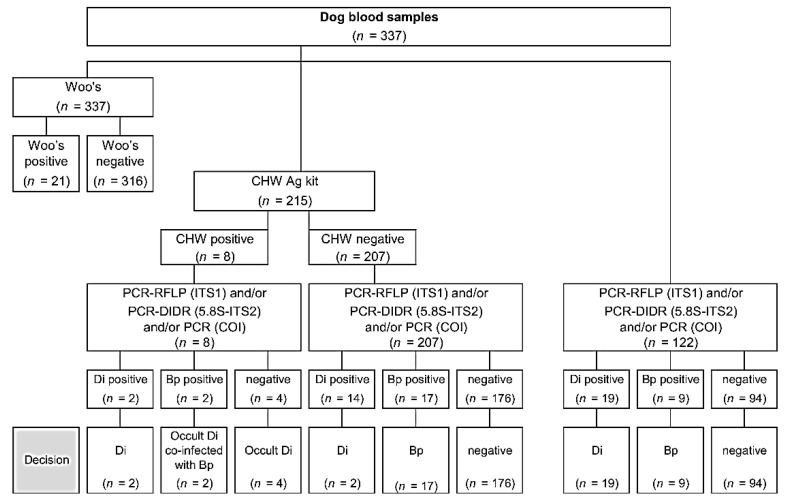
Diagnostic diagram of canine filariasis using CHW Ag test kit and three molecular techniques: PCR-RFLP of ITS1 region, PCR of 5.8S-ITS2-28S region, and PCR of *cox*1 gene. Di: *D. immitis*; Bp: *Brugia pahangi*.

Diagnostic steps for identification and determination of the prevalence of filariasis are presented in Figure 2. The determination of the presence of filariasis was based on three diagnostic techniques: (1) *D. immitis* infection when positive with CHW Ag kit and/or *D. immitis*-DNA-positive with any molecular technique; (2) occult *D. immitis* infection when positive with CHW Ag kit but negative with any PCR; (3) *B. pahangi* infection when *B. pahangi*-DNA-positive with any molecular technique. Descriptive statistics summarizing the prevalence of filarial worm infection are presented as a percentage of each species. Differences in prevalence between groups or categories were analyzed using proportion tests. All statistical analyses were performed using R software [22] and *p*-values < 0.05 were considered statistically significant.

#### 2.3.2. Sequencing and Phylogenetic Analyses

The obtained sequences were compared with the previously deposited ones in GenBank using the BLASTn program [23] for specific diagnosis. Seven successful 5.8S-ITS2 nucleotide sequences originating from two *D. immitis* isolates (Di CM329 and CM331) and five isolates of *B. pahangi* (Bp-CM22, CM188, CM189, CM328, and CM337) (Table S1) were used to analyze the phylogenetic relationship among the regions. In addition, the available sequences of *D. immitis* and *B. pahangi* from other countries (Brazil, Bulgaria, China, India, Iran, Lithuania, Portugal, Taiwan, Thailand, Turkey, Tunisia, and the U.S.; Table S1) were compared with the sequences obtained in this study for the construction of a phylogenetic tree. The sequences of *B. malayi, Onchocerca volvulus, A. reconditum* (previously *Dipetalonema reconditum*) and *D. repens* (GenBank accession: EU373624, EU272179, AF217801, and MK942385, respectively), and the sequence of *Setaria digitata* (EF196091) as the outgroup were included for evolution analysis. All sequences obtained in this study were submitted to the DDBJ/EMBL/GenBank database (Table S1).

Evolutionary analyses were conducted using MEGA X [24]. Multiple sequences were aligned using ClustalW and a phylogenetic tree using a maximum likelihood (ML) method based on the Tamura–Nei model [25]. The Neighbor-Join and BioNJ algorithms were applied to a matrix of pairwise distances estimated using the Tamura-Nei model, and then the topology was selected with superior log likelihood value. A consensus tree was obtained after bootstrap analysis of 100 replications. The tree is drawn to scale, with branch lengths indicating the number of substitutions per site. Thirty nucleotide sequences were included in this analysis. Codon positions included were first, second, third, and noncoding. 

#### 2.3.3. Geographical Information System Mapping and Distribution by Altitude

Geographical Information System (GIS) locations (latitude, longitude, and altitude) from a mobile phone application were input to a computer. The digital elevation model (DEM) was obtained from the NASA Earth data website and used to create a raster image and to convert the model into contour lines representing altitude in the GIS application (QGIS software version 3.6, GNU General Public License). The altitudes of the infected sites were measured using a GPS. Altitudes of the positive prevalence spots were divided into three classes modified from those used by Devi and Jauhari [26]: below 400, 400–800, and over 800 m. A proportional test was used to determine differences in prevalence of *B. pahangi* and *D. immitis* among altitude classes using R software [22] and *p*-values < 0.05 were considered statistically significant. Geographic distribution by altitude was generated from geographic information systems and GPS tracking data using QGIS software version 3.6. Notably, NASA Earth data indicates that all NASA data are available without restrictions.

#### 2.3.4. Geographic Spatial Cluster Analysis

Spatial cluster analyses of the positive *D. immitis* and *B. pahangi* cases were attained using a Bernoulli model and SaTScan™ v9.6 software [27]. Generally, this model requires data from cases and controls. The definition of a case was a positive filarial nematode using any of the three diagnostic techniques (Figure 2), and a negative individual was the control. In the study area, 50% of the total population was set up in the spatial scanning window. The Monte Carlo hypothesis testing technique (number of replications = 999) was used to determine the statistical significance of the cluster. The primary clusters of *D. immitis* and *B. pahangi* were classified based on the highest log-likelihood ratio (LLR), in which the likelihood function of the Bernoulli model is:

(1) $${\left(\frac{c}{n}\right)}^{c}{\left(\frac{n-c}{c}\right)}^{n-c}{\left(\frac{C-c}{N-n}\right)}^{C-c}{\left(\frac{\left(N-n)-(C-c\right)}{N-n}\right)}^{\left(N-n)-(C-c\right)}I(),$$

where C is the total number of cases, N is the combined total number of cases, the control c is the observed number of cases within the window, n is the total number of cases and controls within the window, and I() is the indicator of function I. As the analysis was only focused on detecting clusters with higher than expected rates, I() was set to 1 [27]. The illustrated spatial cluster of each species combined with the altitude and the geographic functional land use was generated by QGIS software version 3.6.

## 3. Results

### 3.1. Prevalence of Filariasis Using a Combination of Conventional and Molecular Techniques

Various diagnostic techniques were employed to identify filarial species infection and molecular techniques were applied to identify the species of filarial nematodes. Of the total of 337 blood samples, microfilaria was detected in only 21 dogs (6.23%) using Woo’s method and most of those were from the central zone (Table 2). Of the 215 blood samples examined using a CHW Ag kit, only eight dogs (3.72%) were *D. immitis* antigen positive (Figure 2). Only three CHW Ag-positive dogs had microfilaremia, of which molecular techniques could discriminate one positive with *D. immitis* and two positives with *B. pahangi*. The overall prevalence of *D. immitis* infection was 12.17% (41/337 dogs), higher than the prevalence of 8.31% for *B. pahangi* (28/337 dogs). However, the difference in infection rate between the two species was not significant (*p* > 0.05). The distributions of *D. immitis* and *B. pahangi* infection in the three zones of Chiang Mai province are shown in Table 2, Table S2, and Figure 1B. The observed *D. immitis* and *B. pahangi* infection incidence was highest in the central zone at 22.96% (31/135), followed by the south zone at 22.45% (22/98). However, the prevalence rates of *D. immitis* and *B. pahangi* infection among the three zones were not significantly different (*p* > 0.05). Two dogs in the central zone had dual infections with *D. immitis* and *B. pahangi*. No adult *D. immitis* antigens were detected in the two *D. immitis* DNA-positive samples. Finally, an important proportion of occult heartworm infections (6/8; 75%) was distinguished (Figure 2).

The range of the percent PCV (%PCV) of dogs with positive microfilariae was 15–46%, with a mean ± SD of 32.48 ± 8.38 and a mode of 31%. Fourteen dogs with positive microfilariae (71.43%; 15/21) had a %PCV lower than 37%, which is considered anemic. Anemic status was categorized as mild (30–37% PCV; 9/21, 42.86%), moderate (20–29% PCV; 4/21, 19.05%), and severe (13–19% PCV; 2/21, 9.52%). No icteric plasma was observed in any of the samples.

### 3.2. Phylogenetic Relationship of D. immitis and B. pahangi

The nucleotide sequences of the partial 5.8S rRNA and ITS2 region contained approximately 300–350 bp that allowed the filarial nematodes to be classified as *D. immitis* or *B. pahangi*. The nucleotide sequences of five isolates of *B. pahangi* were identical and grouped in a haplotype that showed 100% identity with *B. pahangi* (AY988600; [3]) and 95.67% with isolates from cats from the Narathiwat province in Southern Thailand (EU373655; [28]). However, two nucleotide sequences of *D. immitis* had some nucleotide differences, which showed 95–100% identity with the *D. immitis* references (Table S1). The nucleotide sequences of *D. immitis* and *B. pahangi* in this study were deposited in the DDBJ/EMBL/GenBank database, including *D. immitis* (accession No. LC554219-554220) and *B. pahangi* (accession No. LC554214-554218 (Table S1)).

**Figure 3:**
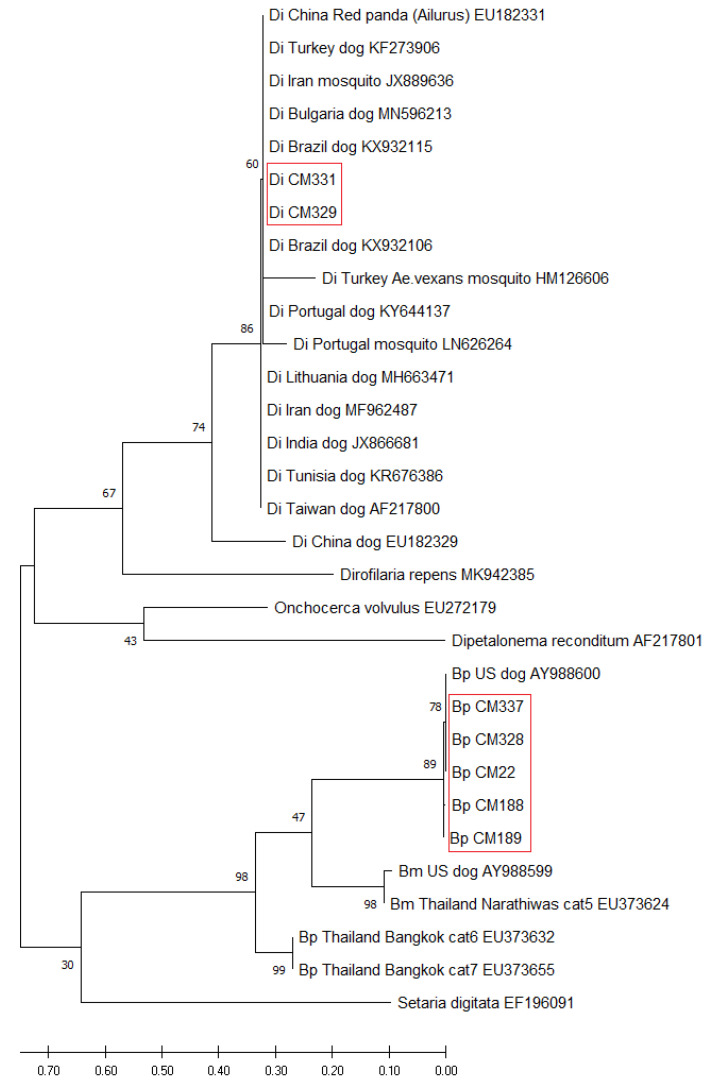
Evolutionary analysis of *D. immitis* and *B. pahangi* 5.8S-ITS2 region. A phylogenetic tree based on the 5.8S-ITS2 region of *D. immitis* (Di) and *B. pahangi* (Bp) from Thailand and other countries was inferred using the maximum likelihood method and the Tamura–Nei model. The tree with the highest log likelihood (−2536.04) is shown. The percentage of trees in which the associated taxa clustered together is shown next to the branches. There was a total of 344 positions in the final dataset. Sequences in the red outline boxes originated from the present study.

A comparative genomic analysis of *D. immitis* and *B. pahangi* was performed to investigate the phylogenetic relationship among the different geographic regions (Figure 3). The phylogenetic tree based on the partial 5.8S and ITS2 sequence showed that all isolated samples from *D. immitis* clustered together in one group and were similar to *D. immitis* from China (EU182331), Brazil (KX932106), Iran (JX889636), Bulgaria (MN596213), and Turkey (KF273906 and HM126606). Five isolates from *B. pahangi* were clustered together in the same clade with *B. pahangi* published by Rishniw et al. [3], in which the worm was from an unknown region. Comparison of nucleotide sequences with other species of filarial nematode found *D. immitis* and *B. pahangi* could be distinguished from *B. malayi*, *D. repens, O. volvulus,* and *A. reconditum* (*Dipetalonema reconditum* in Figure 3).

### 3.3. Geographical Distribution of Filarial Infection

#### 3.3.1. Altitudinal Distribution of Filarial Infection

**Figure 4:**
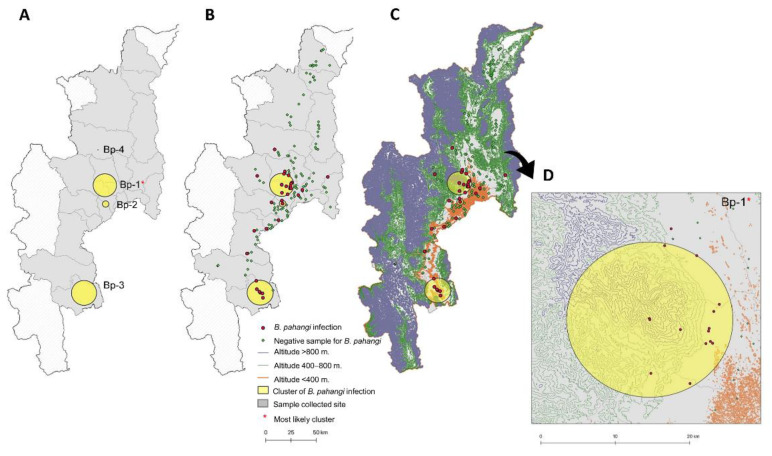
Geographic spatial clusters of *B. pahangi* infection obtained using the Bernoulli scan statistic model. (**A**). The distribution of four clusters of *B. pahangi* infection; (**B**). the distribution of *B. pahangi* cases in each cluster; (**C**). the altitudinal distribution of *B. pahangi* cases in each cluster; (**D**). the distribution of *B. pahangi* cases in the most likely cluster.

**Figure 5:**
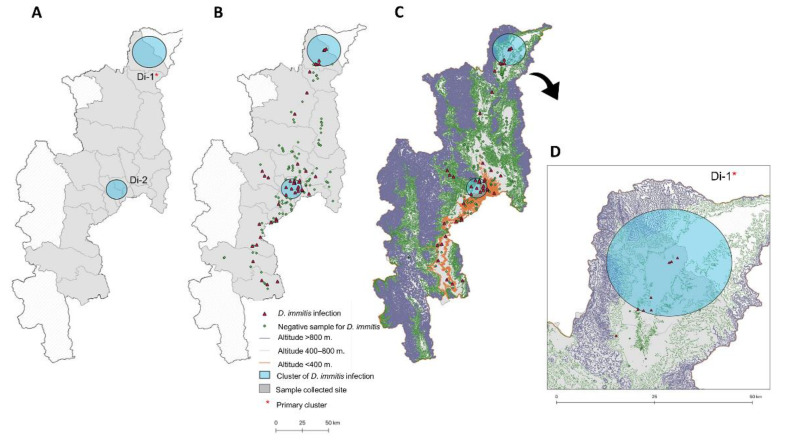
Geographic spatial clusters of *D. immitis* infection obtained using the Bernoulli scan statistic model. (**A**). the distribution of four clusters of *D. immitis* infection; (**B**). the distribution of *D. immitis* cases in each cluster; (**C**). the altitudinal distribution of *D. immitis* cases in each cluster; (**D**). the distribution of *D. immitis* cases in the most likely cluster.

There was no difference in the filarial positivity of either *B. pahangi* or *D. immitis* among the different altitude classes (Table 3). The range of *B. pahangi* infection was 4.08–10.27%, with the highest occurrence observed in the low areas <400 m altitude (10.27%; 23 cases). The range of *D. immitis* infection was 12.05–14.29%, with the highest occurrence observed at 400–800 m (14.29%; 14 cases). The prevalence of *D. immitis* was significantly higher than *B. pahangi* in the middle altitude class of 400–800 m, at 14.29% and 4.08%, respectively (*p* < 0.05; Table 3). The altitudinal distribution of filarial positive cases is illustrated on a GIS map of Chiang Mai, Thailand (Figure 4C and Figure 5C).

#### 3.3.2. Spatial Cluster Distribution of Filarial Infection

The canine filariasis spatial clusters were analyzed using the Bernoulli special scanning statistic method.Tthe relative risk (RR) and size of each cluster are outlined in Table 4. Four and two spatial clusters were identified for *B. pahangi* and *D. immitis*, respectively. Regarding *B. pahangi* distribution, a significant cluster (cluster Bp-1; *p* < 0.001) was identified with RR at 6.69 and a clustering radius of 10.55 km. This most likely cluster was distributed in three districts: Hang Dong, Mae Rim, and Mueang. The primary cluster of *B. pahangi* infection was found to occur in various altitudinal areas (0 to >800 m Figure 4C,D). Replacement of forests by agricultural activities and irrigated crops occurred in scattered locations in this cluster area. However, no significant spatial patterns were identified for *D. immitis* infection (Table 4), although the number of cases observed was higher than with *B. pahangi*. The most likely cluster of *D. immitis* infection (Di-1) was located in the Fang district (18.737609° N, 98.931619° E) where the clustering radius was 9.32 km, and the RR was 4.03. Sites of positive identification of *D. immitis* in this cluster were distributed in the low areas at altitudes of 400 to 800 m (Figure 5C,D) and were found mainly in areas with irrigated agricultural crops.

## 4. Discussion

The current study updates our understanding of the situation of *D. immitis* infection and proven *B. pahangi* infection in community dogs in Northern Thailand. *B. malayi* was not found in Chiang Mai, although the province does have a large free-roaming dog population.

Heartworm disease caused by *D. immitis* infection is widespread in tropical and subtropical regions and is a primary life-threatening disease of dogs and cats worldwide. The prevalence of *D. immitis* infection in dogs worldwide was 10.91% (95% CI = 10.18–11.65) in 2020. The prevalence of *D. immitis* in dogs varies across countries and continents, e.g., 22.68% in Australia, 12.07% in Asia, 11.60% in the Americas, 10.45% in Europe, and 7.57% in Africa [1]. Over the past few decades, the regional prevalence of *D. immitis* infection in dogs in Thailand has been reported as 10–58% in Bangkok [16,29,30,31], 23–25% in the southern region [32], and 6–25% in the northern region [19,33,34,35]. In Chiang Mai, the prevalence gradually decreased from 45.76% in 1987 to 24.71% in 1992, to 18.20% in 2008, and then to 12.17% in 2020. The reduction of dirofilariasis is possibly due to effective regular preventive chemotherapy of dogs and to vector control by dog owners in collaboration with the active support of District Administration Organizations, which should be extended to other high prevalence districts such as Fang.

*B. pahangi,* a lymphatic filarial worm that infests mammals, is commonly found in cats, dogs, and wild carnivores [36], and is closely related to *B. malayi*. Recently, five cases of clinically typical LF caused by *B. pahangi* were reported in suburban areas in the city of Kuala Lumpur, Malaysia [9]. Additionally, there was a report of a human subconjunctival infection with *B. pahangi* in Malaysia, which was identified by cyclooxygenase-1 (COX-1) PCR [37]. To the best of our knowledge, that study was the first to confirm *B. pahangi* infection in an animal in Chiang Mai. Elsewhere in Thailand, the *B. pahangi* infection rate range of 4–25% in dogs and cats in Bangkok has been reported [16,31,38]. In addition, in Western and Southern Thailand, accidental zoonotic filariasis of *B. pahangi* infections in children were reported, and *Armigeres subalbatus* was found to be a common mosquito in the infected areas [39]. The recently discovered parasite *B. pahangi* exhibits novel aspects and adaptations. For that reason, *Brugia* infections in humans in this region should be closely monitored, especially the environment and geographical distribution in areas with a high vector density and densely crowded human populations.

Natural infections of canine dirofilariasis enhance the risk of transmission to humans [40]. The relative risk of canine dirofilariasis has been found to be related to various factors, e.g., outdoor living, lack of heartworm prevention, and infected vector exposure [19,40]. The present study observed *D. immitis* cases in all altitude classes (0 to >800 m), probably due to the broad range of mosquito species, which are potential transmission vectors. Although *Mansonia uniformis* is an important natural mosquito vector of *D. immitis* in Chiang Mai [33], *Culex pipiens quinquefasciatus*, *Aedes albopictus*, and *Ae. aegypti* are additional vector species for *D. immitis* transmission. The majority of *Aedes*, *Anopheles*, *Armigeres,* and *Culex* mosquitoes have been found at altitudes between 300 and 900 m [26]. *Ae. albopictus* is distributed in a wide range of elevations, between 300 and 1300 m, whereas *Cx. quinquefasciatus* has been found at elevations of 500 m and above [26] and *Mansonia* species are spread across different altitudes [41]. The number of *Mansonia* species is likely to be higher in areas with floating aquatic plants.

*B. pahangi* is considered to be a new causative agent of urban *Brugia* filariasis in Thailand [42]. Infective-stage larvae of *B. pahangi* microfilariae can develop in *An. quadrimaculatus* and *Ae. aegypti* [43]. Development and transmission of *B. pahangi* microfilariae to a vertebrate host can occur via natural vectors such as *Ar. subalbatus* [44,45]. *Ar. subalbatus* has also been found to be a common mosquito in infected areas and is suspected of being the main vector for zoonotic *B. pahangi* infection of children in Thailand [39]. Chaves et al. [46] demonstrated that density of adult *Ar. subalbatus* decreases with altitude. Its preferred range is 109–330 m, but it tends to increase in areas with abundant leaf litter on the ground. However, other mosquitoes, including the genera *Culex*, *Aedes*, *Mansonia*, *Anopheles,* and *Armigeres*, are potential mosquito vectors of *Brugia* spp. [47] which might explain the distribution of *B. pahangi* cases at all altitudes from 0 to >800 m. Climatic and geographic conditions, as well as environmental factors and changes in land use, may support the high prevalence of *B. pahangi* at low altitudes. The occurrence of more hotspots of *D. immitis* and *B. pahangi* in suburban areas of Chiang Mai, such as the Fang and Mae Rim districts (Figure 4), could be influenced by factors driving transmission such as urbanization, rural–urban migration, and expansion of irrigated agricultural land, which is common in this area and provides suitable humidity for mosquitoes to thrive. Spatial mapping of the mosquito vector distribution and breeding habitats in canine filarial-infected areas on finer scales should be conducted to complete our current understanding of transmission dynamics and to help with the establishment of appropriate control strategies.

Although results of analyses of the correlation among diagnostic techniques for filariasis have so far been inconclusive [48,49], a combination of various diagnostic methods remains crucial for precise canine filariasis mapping of the region. Although the sensitivity of microhematocrit centrifugation for microfilaria detection was reported to be only 30% [50], this concentration technique is commonly applied in clinical practice to screen for microfilaria prior to confirmation using a heartworm antigen kit. We did not differentiate the microfilaria species by microscopic examination because this is labor-intensive and because of the difficulty in resolving the identification of species using blood smears. However, molecular techniques such as PCR can successfully detect microfilaria from blood samples. Additionally, PCR can confirm species identification using specific primers or by sequencing, making it more precise than microscopic procedures, especially when many filarial species and coinfection are involved. In the present study, two *D. immitis* DNA-positive dogs did not present with adult *D. immitis* antigen in their blood. This may be linked to the existence of an immune complex that blocks the antigen detection process or to the low antigen secretion by adult worms [51]. However, one occult infection was apparently observed in the current study. This detection problem might be due to many factors, including detection in dogs with prepatent infection, immature female worms, unisexual worm infection, drug-induced sterility of adult heartworms, senile infertility of female worms, host immune responses, and treatment with macrocyclic lactones [6,52,53].

This study has some limitations. First, although we covered a wide range of geographical features in Northern Thailand, the results should be interpreted with caution as they cannot be generalized to all endemic settings. More variations in geographical characteristics should be included in future studies. Second, as sampling was conducted mainly among free-roaming dogs in the studied communities, information about the dogs, e.g., housing, routine drugs, and vaccination, was limited; thus, an analysis of risk factors associated with the risk of *B. pahangi* could not be determined in this study. Last, the sampling strategy in this study was from an unknown prevalence; thus, it may pose a challenge, especially for *B. pahangi*, possibly contributing to an underestimation of number of samplings. However, for a follow-up study, the prevalence obtained from this study might be used and modified for a sample size determination.

## 5. Conclusions

In this study, we covered the detection and confirmation of species of canine filarial infection in free-roaming community dogs found in Chiang Mai, Northern Thailand, using a combination of diagnostic methods. The infection rates of *B. pahangi* were found to be comparable to those of *D. immitis*. Both types of filarial nematodes had a high prevalence in the low altitude suburban agricultural areas. The residents of the region should be made aware that lymphatic filariasis is caused by *B. pahangi*. For practicing veterinarians, filarial species identification is necessary for determination of the proper heartworm treatment as well as for public health efforts related to zoonotic transmission. Spatial distribution of filariasis in consecutive areas as well as mosquito vector distribution and breeding habitats of *D. immitis* and *B. pahangi* in infected areas should be determined at finer scales to identify and implement appropriate control strategies.

## Figures and Tables

**Table 1:** Primer pairs used for PCR amplification.

Gene Target	Primer Pairs	Primer Sequence (5′-3′)	Filarial Species	Product Size (bp)
ITS1	ITS1-F ITS1-R	GGT GAA CCT GCG GAA GGA TC GAG TTA CGC AGA CGT TAA GCG	*W. bancrofti*	482
			*B. malayi*	504
			*B. pahangi*	510
			*D. immitis*	595
			*D. repens*	602
5.8S-ITS2-28S	DIDR-F1 DIDR-R1	AGT GCG AAT TGC AGA CGC ATT GAG AGC GGG TAA TCA CGA CTG AGT TGA	*D. immitis*	542
			*A. reconditum*	578
			*D. repens*	484
			*A. dracunculoides*	584
			*B. pahangi*	664
			*B. malayi*	615
COI	DI COI -F1 DI COI-R1	AGT GTA GAG GGT CAG CCT GAG TTA ACA GGC ACT GAC AAT ACC AAT	*D. immitis*	203

**Table 2:** Prevalence of microfilariae and filarial nematode species in the community dogs among three zones of Chiang Mai, Thailand.

Zone	No. of Examined	Microfilariae (Woo’s Method)	Filarial DNA (Molecular Techniques)			
		% Positive (no.)	*B. pahangi* Infection (*n1*)	*D. immitis* Infection (*n2*)	Dual Infection (*n3*)	Total (*n1 + n2 − n3*)
North	104	2.88 (3) ^a, b^	3.85 (4)	9.62 (10)	0	13.46 (14)
Central	135	11.11 (15) ^b^	10.37 (14)	14.07 (19)	2	22.96 (31)
South	98	3.06 (3) ^a^	10.20 (10)	12.24 (12)	0	22.45 (22)
Total	337	6.23 (21)	8.31 (28)	12.17 (41)	2	19.88 (67)

^a, b^ values in the same column with different superscripts are statistically different (*p* < 0.05); *n1*, *n2*, *n3* are the number of positive cases.

**Table 3:** The altitudinal distribution and prevalence of canine filariasis in Chiang Mai, Thailand.

Level	Altitude Level (m.)	No. of Examined	*B. pahangi* Infection (*n*)	*D. immitis* Infection (*n*)	Total Canine Filariasis (*n*)
1	<400	224	10.27% (23)	12.05% (27)	21.43% (48)
2	400–800	98	4.08% (4) ^a^	14.29% (14) ^b^	18.37% (18)
3	>800	15	6.67% (1)	0	6.67% (1)
Total		337	8.31% (28)	12.17% (41)	19.88% (67)

^a, b^ Values in the same column with different superscripts are statistically different (*p* < 0.05).

**Table 4:** Geographic spatial clusters obtained by Bernoulli scan statistic model on canine filariasis in Chiang Mai, Thailand.

Cluster Number	Cluster Type	Centroid (X,Y)/Radius (km)	Cases	Expected Cases	Observed to Expected Cases	RR ^a^	LLR ^b^	*p*-Value	Districts
*B. pahangi* infection									
Bp-1	Most likely	18.815706 N, 98.883035 E/10.55	8	1.58	5.07	6.69	8.848	0.010	Hang Dong, Mae Rim, Mueang, Samoeng
Bp-2	Secondary	18.660241 N, 98.889764 E/2.91	2	0.17	12.04	12.88	5.043	0.439	Hang Dong, San Pa Tong
Bp-3	Secondary	17.924869 N, 98.704168 E/11.42	5	1.41	3.54	4.09	3.457	0.754	Doi Tao
Bp-4	Secondary	19.108108 N, 98.824182 E/0	2	0.25	8.02	8.56	3.214	0.883	Mae Taeng
*D. immitis* infection									
Di-1	Most likely	18.737609 N, 98.931619 E/9.32	9	2.68	3.36	4.03	6.386	0.143	Fang
Di-2	Secondary	19.922648 N, 99.208706 E/15.01	6	1.70	3.52	3.96	4.392	0.393	Hang Dong, Mueang, Saraphi

^a^ RR = relative risk, ^b^ LLR = log likelihood ratio.

## Data Availability

Data available on request due to privacy for sequential projects.

## Supplementary Materials

The following are available online at https://www.mdpi.com/2076-2615/11/1/33/s1, Table S1: The 5.8S-ITS2 sequence list of *D. immitis, B. pahangi,* and other filarial nematodes used in the phylogenetic analysis, Table S2: Prevalence of canine filariasis in different districts in Chiang Mai Province, Thailand.
