# Supplementary material for: Geographic Spatial Distribution Patterns of Dirofilaria immitis and Brugia pahangi Infection in Community Dogs in Chiang Mai, Thailand
Source: Animals (Basel). 2020 Dec 26;11(1):33. doi: 10.3390/ani11010033 (PMC7824140; doi:10.3390/ani11010033)
Supplement: Supplementary file 1 [file animals-11-00033-s001.pdf]

animals

MDPI

Table S1. The 5.8S-ITS2 sequence list of D. immitis, B. pahangi, and other filarial nematodes used for phylogenetic analysis.

Filaria Species and Isolates	Accession No.	References
D. immitis		
Di_CM329	LC554219	This study
Di_CM331	LC554220	
Di_Brazil_dog	KX932115	[1]
Di_Brazil_dog	KX932106	
Di_Bulgaria_dog	MN596213	[2]
Di_Lithuania_dog	MH663471	[3]
Di_Portugal_dog	KY644137	[4]
Di_Portugal_mosquito	LN626264	[5]
Di_Iran_dog	MF962487	[6]
Di_Iran_mosquito	JX889636	[7]
Di_India_dog	JX866681	[8]
Di_China_Red panda (Ailurus)	EU182331	[9]
Di_China_dog	EU182329	
Di_Tunisia_dog	KR676386	[10]
Di_Taiwan_dog	AF217800	[11]
Di_Turkey_dog	KF273906	[12]
Di_Turkey_Ae.vexans mosquito	HM126606	
Brugia		
Bp_CM22	LC554214	This study
Bp_CM188	LC554215	
Bp_CM189	LC554216	
Bp_CM328	LC554217	
Bp_CM337	LC554218	
Bp_US_dog	AY988600	[13]
Bp_Thailand_Bangkok_cat7	EU373655	[14]
Bp_Thailand_Bangkok_cat6	EU373632	
Bm_Thailand_Narathiwas_cat5	EU373624	
Bm_US_dog	AY988599	[13]
Other Filarial nematodes		
Onchocerca volvulus	EU272179	[15]
Dipetalonema reconditum	AF217801	[11]
Setaria digitata	EF196091	[16]
Dirofilaria repens	MK942385	[17]

Table S2. Prevalence of canine filariasis in different districts in Chiang Mai, Thailand.

Collecting sites	Districts	*n*	*B. pahangi*	*D. immitis*
			No. (%)	No. (%)
Northern zone	Mae Rim	17	2 (11.76)	0
	Mae Tang	18	2 (11.11)	0
	Phrao	17	0	0
	Chai Prakan	17	0	1 (5.88)
	Chiang Dao	16	0	3 (18.75)
	Fang	19	0	6 (31.58)
Central zone	Mae On	8	1 (12.50)	0

	San Kamphaeng	14	1 (7.14)	1 (7.14)
	Doi Saket	12	0	1 (8.33)
	Saraphi	15	1 (6.67)	4 (26.67)
	San Sai	14	0	2 (14.29)
	Samoeng	11	1 (9.09)	3 (27.27)
	Mae Wang	13	1 (7.69)	1 (7.69)
	San Pa Tong	19	1 (5.26)	0
	Mueang*	21	6 (28.57)	6 (28.57)
	Hang Dong*	8	2 (25)	1 (12.5)
Southern zone	Doi Lo	26	3 (11.54)	3 (11.54)
	Chom Thong	22	2 (9.09)	4 (18.18)
	Hot	24	0	1 (4.17)
	Doi Tao	26	5 (19.23)	4 (15.38)
Total		337	28 (8.31)	41 (12.17)

* one sample was positive PCR of both species

.
